# Postburn Downregulation of miR‐148a‐3p and Its Regulatory Mechanism in Hypertrophic Scar Formation

**DOI:** 10.1111/jocd.71174

**Published:** 2026-09-11

**Authors:** Yanhua Lu, Meng Yu, Jinghe Zhou, Yang Yang

**Affiliations:** ^1^ Department of Burn and Plastic Surgery The Second Affiliated Hospital of Shandong First Medical University Tai'an China; ^2^ Burn, Plastic and Microsurgery Department The Fourth Affiliated Hospital of China Medical University Shenyang China; ^3^ Medical Aesthetics Department Affiliated Hangzhou First People's Hospital, School of Medicine, Westlake University Hangzhou China; ^4^ Division of Trauma and Acute Care Surgery, Department of Emergency Medicine Jinling Hospital, Affiliated Hospital of Medical School, Nanjing University Nanjing China

**Keywords:** hypertrophic scar, MEOX2, miR‐148a‐3p, skin fibroblast

## Abstract

**Background:**

Burn injuries represent a common form of trauma, and their healing process poses a significant clinical challenge, frequently complicated by aberrant scarring. Recent studies have found that miR‐148a‐3p is an important regulator involved in the process of tissue fibrosis, but the specific mechanism of action of miR‐148a‐3p in burn wound repair still needs to be elucidated.

**Methods:**

Clinical data were collected from participants, and serum miR‐148a‐3p expression was measured by qRT‐PCR in both burn patients and healthy controls. ROC curve analysis and logistic regression were employed to evaluate hypertrophic scar occurrence at 6‐month follow‐up. In vitro, a heat‐injured fibroblast model was established. The cellular proliferation, migration, and expression of fibrosis markers (Col I, α‐SMA, TGF‐β1) were assessed using CCK‐8 assay, Transwell migration assay, and qRT‐PCR. The targeting relationship between miR‐148a‐3p and MEOX2 was predicted by bioinformatics analysis and verified by dual‐luciferase reporter assay. Functional rescue experiments were performed to confirm the regulatory mechanism.

**Results:**

Compared to healthy subjects, miR‐148a‐3p expression was significantly lower than that of the healthy control group, and the reduction was more pronounced in patients with scarring, regardless of age, weight, or sex. The expression level of miR‐148a‐3p and the depth of burn injury constitute important risk factors for scarring, which has significant diagnostic value. The expression level of miR‐148a‐3p in human skin fibroblasts (WS1) decreased significantly after heat treatment compared with that before treatment. Overexpression of miR‐148a‐3p inhibited the proliferation and migration of WS1 cells and downregulated the expression of fibrosis‐related markers such as collagen I, α‐SMA, and TGF‐β1. Mechanistic studies have shown that miR‐148a‐3p negatively regulates its expression by directly binding to MEOX2, and overexpression of MEOX2 can reverse the cell function and other disorders caused by miR‐148a‐3p.

**Conclusion:**

miR‐148a‐3p plays a key role in postburn hypertrophic scarring by targeting MEOX2 to affect fibroblast activity, suggesting its potential value as a diagnostic marker and a target for scar prevention and treatment.

## 1 Instruction

Burns are a common type of trauma in clinical practice, and the wound healing process is often accompanied by pathological scarring, which severely affects the patient's appearance and functional recovery, and eventually forms hypertrophic scars (HS) [1]. HS is a fibrotic disorder caused by abnormal wound healing resulting in increased collagen synthesis and deposition, a common complication after burns and trauma [2]. It is often associated with pain, itching, and limited joint motion, and in severe cases, can even affect the patient's quality of life [3]. Although there have been significant advances in burn treatment technology in recent years, scar hyperplasia is still a major clinical challenge.

MicroRNAs (miRNAs) are a class of endogenous, non‐coding, single‐stranded small RNAs that regulate the expression of genes and proteins at the posttranscriptional level by binding to target genes in a completely or incompletely complementary manner [4]. There is evidence that miRNAs play a key role in the scarring process, and miR‐21, miR‐155, miR‐96, etc., promote collagen deposition by activating signaling pathways such as TGF‐β/Smad, thereby accelerating keloid formation [5, 6, 7]. Existing studies have shown that miR‐148a‐3p, as a highly conserved miRNA in mammals, shows a significant down‐regulation trend in a variety of fibrotic diseases [8]. The miRNA was significantly downregulated in a variety of diseases, including liver fibrosis and pulmonary fibrosis, and may form a complex regulatory network by targeting multiple fibrosis‐related genes such as Cyth3 and β‐catenin [9, 10, 11]. It is important to note that recent studies have confirmed the good diagnostic value of miR‐148a‐3p in keloids [12], but the specific mechanism of action in the process of HS after burns remains to be elucidated. In addition, MEOX2 is an important transcription factor and plays an important role in fibroblast activation and fibrosis as a potential downstream target gene of miR‐148a‐3p [13]. Studies demonstrate MEOX2's involvement in pathological scar formation through dual mechanisms: promoting collagen synthesis while inhibiting matrix degradation [14]. This transcription factor shows strong associations with multiple fibrotic conditions, spanning localized disorders (hypertrophic scars, keloids) to systemic diseases (liver fibrosis, cardiac fibrosis) [15, 16]. MEOX2 is an important molecule in the regulation of tissue fibrosis [15], making it a potential target for antifibrotic therapy, but its specific mechanism of action in the regulatory network of miR‐148a‐3p‐mediated scarring remains to be elucidated.

The purpose of this study was to explore the regulatory role of miR‐148a‐3p in postburn scar formation and its molecular mechanism. It provides a new molecular target for the development of miRNA‐based precision therapy strategies and provides clinical translational value for improving the wound healing quality and alleviating scar hyperplasia in burn patients.

## 2 Materials and Methods

### 2.1 Clinical Samples and Analysis

This study was approved by The Second Affiliated Hospital of Shandong First Medical University Ethics Committee. A total of 72 burn patients were included between January and June 2024, alongside 67 concurrently selected normal subjects as controls, and the baseline data of all subjects were collected. All participants signed an informed consent form. Detailed demographic (age, sex, BMI) and scar‐related parameters (diameter, Vancouver Scar Scale score) were documented. Regarding burn types, the cohort included patients with thermal burns (scald and flame burns) affecting 10%–30% total body surface area, with partial‐ to full‐thickness wounds. Patients with electrical, chemical, or radiation burns were excluded. All patients received standardized local wound care and conservative management, with no systemic agents (e.g., corticosteroids, antifibrotic drugs, or immunosuppressants) administered during the follow‐up period that could interfere with scarring. Serum collection protocol: Venous blood (5 mL) was collected from burn patients within 24 h of admission and from controls during routine physical examinations. All samples were centrifuged at 3000 rpm for 15 min, aliquoted, and stored at −80°C for subsequent testing. Patients were followed for 6 months postinjury, with hypertrophic scar diagnosis confirmed by: (1) persistent erythema/elevation beyond 3 months, (2) histopathological verification when clinically indicated, and (3) ≥ 2 mm elevation on ultrasound imaging.

### 2.2 Cell Culture, Transfection and Modeling

The human dermal fibroblast cell line WS1 was obtained from the American Type Culture Collection (ATCC) and cultured in RPMI 1640 medium supplemented with 10% fetal bovine serum (FBS) and 100 U/mL penicillin–streptomycin at 37°C. When the cells reached 70%–80% confluency, an in vitro burn injury model was established by subjecting them to a 30‐s heat exposure at 52°C. Following this injury, the cells were incubated for 48 h to model the scar formation process. All subsequent analyzes were performed after this 48‐h incubation period, except the proliferation assays. miR‐148a‐3p mimic (miR mimic), miR‐148a‐3p inhibitor (miR inhibitor), pcDNA3.1‐MEOX2 (oe MEOX2) and their negative controls mimic NC, inhibitor NC, pcDNA3.1‐empty (oe NC) were treated with Lipofectamine 2000 (Invitrogen, USA). Follow‐up experiments were performed 48 h after transfection of cells at room temperature.

### 2.3 Real‐Time Quantitative PCR

Tissue and cell samples were mixed with Trizol reagent (Thermo, USA) and allowed to stand on ice for 5 min. Chloroform was then added and centrifuged at 12 000 *g* for 15 min. Take the supernatant and mix with isopropanol and centrifuge at 4°C for 10 min. The pellet was washed with ethanol to obtain total RNA. RNA concentration and purity were assessed using the NanoDrop2000 (Thermo, USA) OD260/280 values, and an OD260/280 ratio between 1.8 and 2.0 indicated good RNA quality. Evo M‐MLV RT Master Mix (Accurate Biology, China) was used to reverse transcribe the extracted RNA into cDNA. Specific primers were used to detect the expression of miR‐148a‐3p, MEOX2, collagen I, α‐SMA, TGF‐β1, and the relative expression levels were calculated by the 2^−ΔΔCt^ method. U6 served as the endogenous control for miR‐148a‐3p, while GAPDH was used as the reference gene for other targets. The primer sequences used were as follows: miR‐148a‐3p forward 5′‐TCAGTGCACAGAACTTTGT‐3′ and reverse 5′‐CAGGTCCAGTTTTTTTTTTTTTT‐3′; U6 forward 5′‐GGGCCATGCTAATCTTCTCTGTA‐3′ and reverse 5′‐CAGGTCCAGTTTTTTTTTTTTTT‐3′; α‐SMA forward 5′‐GTTGCTGCTTGCAGTAACCTT‐3′ and reverse 5′‐AGGGCCAAGTCCAACTCCTT‐3′; MEOX2 forward 5′‐GAGGAAAAGCGACAGCTCAGAT‐3′ and reverse 5′‐TCTCTGATTTGCTCCTTGGTGA‐3′; collagen I forward 5′‐GAGGGCCAAGACGAAGACATC‐3′ and reverse 5′‐CAGATCACGTCATCGCACAA‐3′; TGF‐β1 forward 5′‐CGACTACTACGCCAAGGA‐3′ and reverse 5′‐GAGAGCAACACGGGTTCA‐3′.

### 2.4 CCK8 Assay

Cells from each group were seeded into a 96‐well plate, with three replicates for each group. The cells were cultured for 24, 48, and 72 h, respectively. Add 10 μL of test solution to each well and incubate at 37°C with 5% CO_2_ for 4 h. Measure the absorbance at 450 nm using a microplate reader, with triplicate measurements for each sample.

### 2.5 Transwell Assay

Single‐cell suspensions were prepared in serum‐free DMEM. For Transwell invasion (pre‐coated with Matrigel) and migration (without Matrigel) assays, 150 μL of cell suspension was seeded into the upper chamber, and the lower chamber was filled with 600 μL of DMEM containing 10% FBS to create a chemotactic gradient. After 24 h of incubation, the cells were fixed and stained, and five random fields were counted per membrane. All experiments were performed in triplicate.

### 2.6 Western Blot Analysis

Total protein was extracted from WS1 cells using RIPA lysis buffer containing protease inhibitors. Protein concentrations were determined using a BCA protein assay kit. Equal amounts of protein (30 μg) were separated by 10% SDS‐PAGE and transferred onto PVDF membranes. The membranes were blocked with 5% skim milk in TBST for 1 h at room temperature, then incubated overnight at 4°C with primary antibodies against collagen I, α‐SMA, TGF‐β1, and GAPDH (as internal control). After washing, the membranes were incubated with HRP‐conjugated secondary antibodies for 1 h at room temperature. Protein bands were visualized using an enhanced chemiluminescence (ECL) detection system and quantified using ImageJ software.

### 2.7 Dual‐Luciferase Reporter Assay

A pGL3 luciferase reporter vector containing miR‐148a‐3p and MEOX2 binding sites (wild‐type WT) and mutant (MUT) was constructed. WS1 cells were seeded in 12‐well plates and transfected with the corresponding vehicle and mimetic inhibitors. Forty‐eight hours after transfection, luciferase activity was measured.

### 2.8 Statistical Analysis

SPSS 23.0 was used for statistical analysis. Normally distributed, continuous data were presented as x̄ ± sd, and all experiments were replicated three times independently, using a *t*‐test or one‐way analysis of variance (LSD‐t multiple comparisons) for comparison between groups. Pearson correlation analysis showed the correlation between miR‐148a‐3p and MEOX2 expression; the diagnostic value was evaluated by ROC curve, and the independent risk factors were analyzed by logistic regression analysis. *p* < 0.05 was statistically significant.

## 3 Results

### 3.1 Baseline Characteristics

The baseline data analysis showed that there were no significant differences in age, body mass index (BMI), and gender distribution between the healthy control group and the burn patient group (*p* > 0.05, Table 1). The results of the 6‐month follow‐up showed that the occurrence of scar hyperplasia was significantly correlated with burn depth (*p* = 0.004), but not with burn area, and the incidence of scar hyperplasia was rarely seen in patients with superficial burns, while the incidence of scar hyperplasia was significantly increased in patients with deep burns (Table 2).

**TABLE 1:** Relationship of clinical characteristics between healthy and burn groups.

Index	Burn group (72)	HC group (67)	*t*/*x* ^2^	*p*
Age	48.10 ± 10.83	46.46 ± 10.21	0.914	0.362
BMI	23.05 ± 1.18	23.19 ± 1.19	0.710	0.479
Gender	35/37	31/36	0.076	0.782
Diameter (≥ 5 cm; < 5 cm)	39/33			
Degree (deep second‐degree; superficial second‐degree)	33/39			

**TABLE 2:** Baseline and 6‐month follow‐up characteristics by study group.

Index	SH group (55)	N‐SH group (17)	*t*/*x* ^2^	*p*
Age	47.67 ± 10.62	49.47 ± 11.71	0.604	0.548
BMI	23.12 ± 1.00	22.8 ± 1.63	0.714	0.492
Gender	26/29	9/8	0.224	0.885
Diameter (≥ 5 cm; < 5 cm)	28/27	11/6	0.996	0.318
Degree (deep second‐degree; superficial second‐degree)	20/35	13/4	8.414	0.004

### 3.2 Significance of miR‐148a‐3p in the Diagnosis of Keloids

**FIGURE 1:**
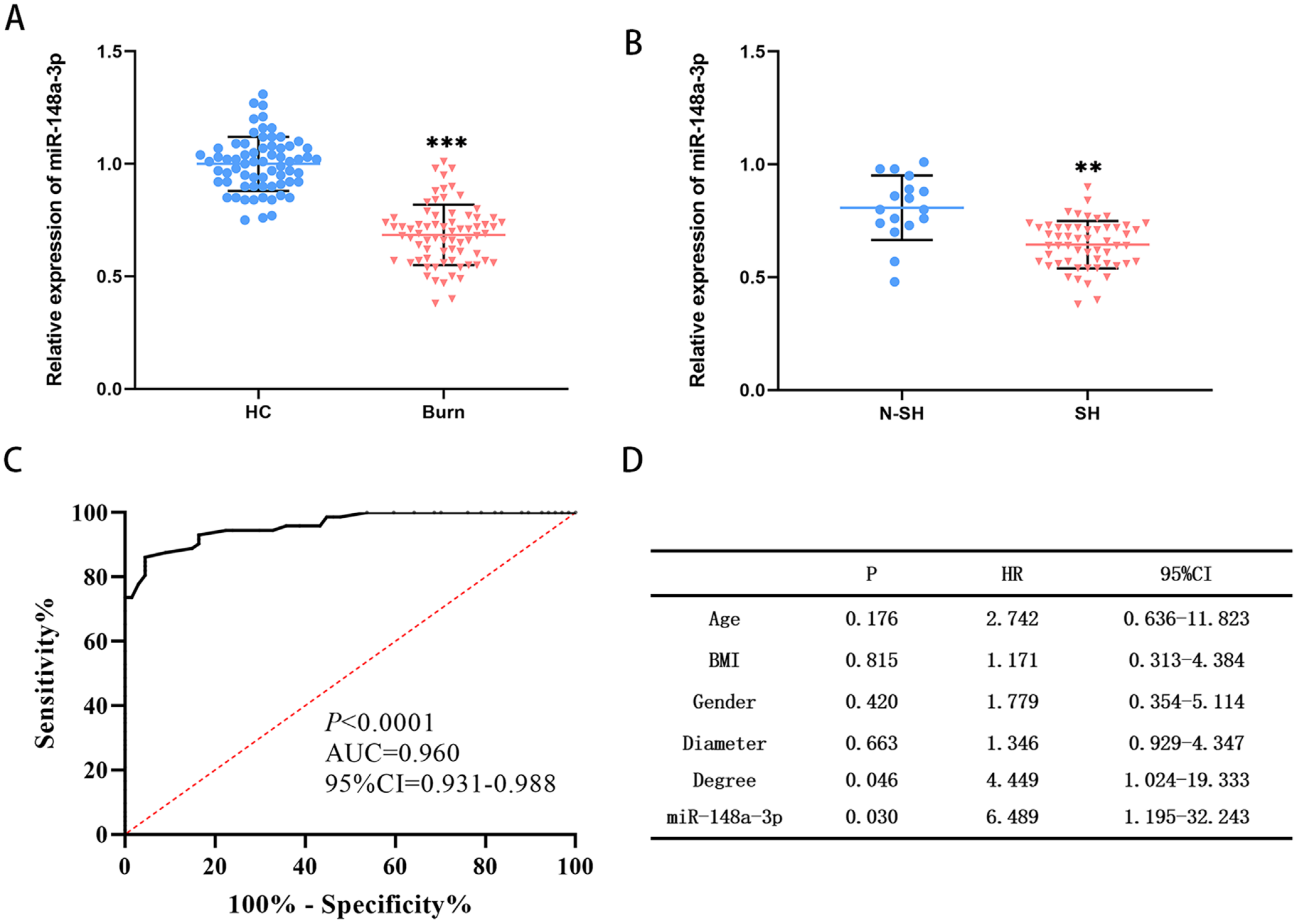
Expression and diagnostic value of miR‐148a‐3p in burn patients. (A) miR‐148a‐3p expression was significantly reduced in burn patients compared to healthy controls. (B) The lowest levels were observed in patients in the HS group compared to the H‐SH group. (C) Logistic analysis showed that miR‐148a‐3p had a strong predictive value (AUC = 0.960). (D) Multivariate analysis confirmed miR‐148a‐3p and burn depth as independent diagnostic factors. (***p < 0.001, **p < 0.01, relative to HC group or N‐SH group by one‐way ANOVA).

Serum molecular marker analysis further revealed that the expression level of miR‐148a‐3p in the serum of burn patients was significantly lower than that in the healthy control (HC) group (Figure 1A), and the expression level of miR‐148a‐3p in the HS group was further downregulated compared with that in the non‐HS (N‐HS) group (Figure 1B). Based on the results of serum analysis, we plotted the patient's diagnostic curve by logistic regression (Figure 1C). The results showed that serum miR‐148a‐3p level had a significant predictive value for scar hyperplasia after burn injury (AUC = 0.960, 95% CI = 0.931–0.988, *p* < 0.0001). At the same time, ROC analysis further determined that miR‐148a‐3p levels were used as a valid predictor of scar hyperplasia (Figure 1D).

### 3.3 Heat Stress Affects Fibroblast Activation and Decreases miR‐148a‐3p Expression

**FIGURE 2:**
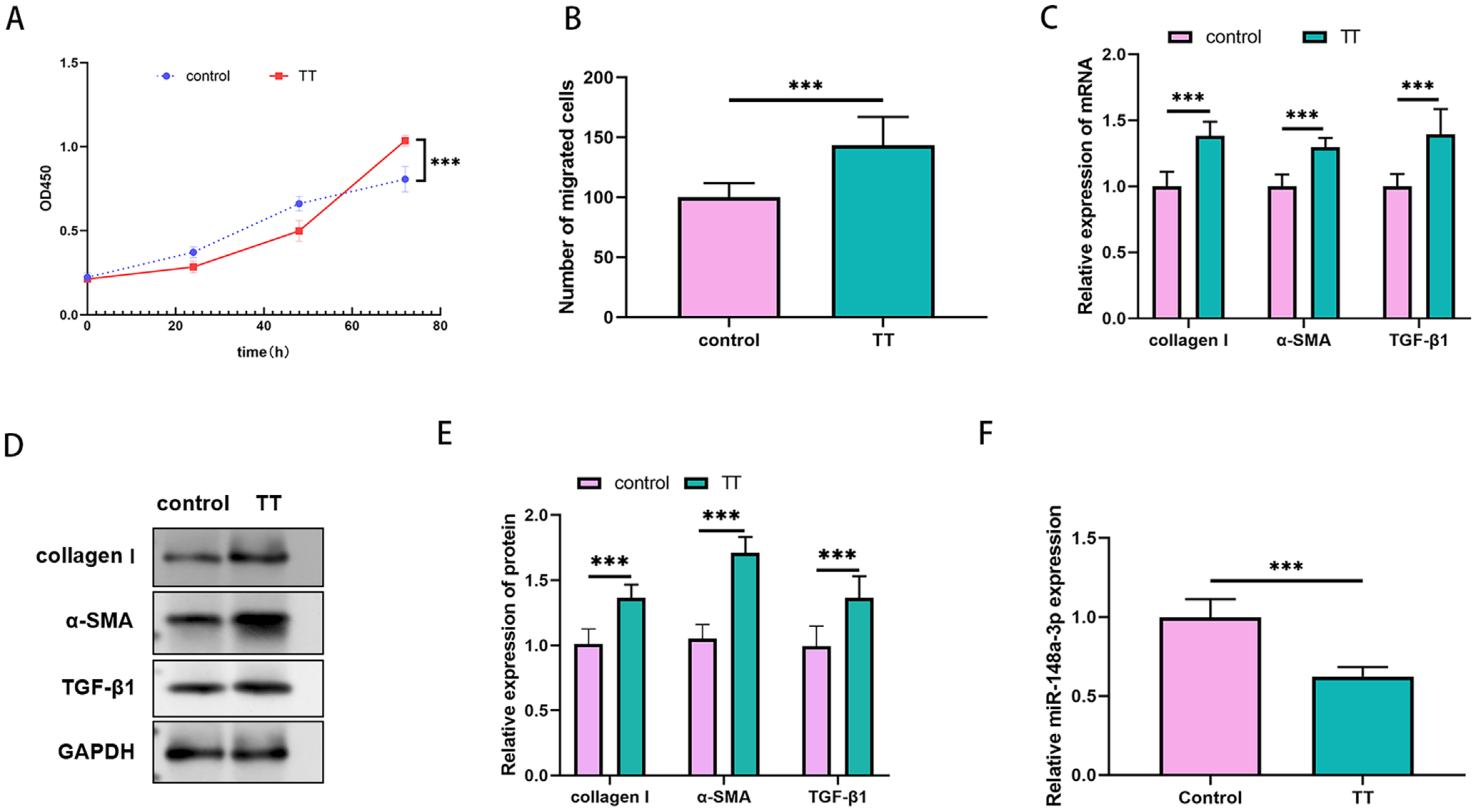
Heat stress induces fibroblast activation and downregulates miR‐148a‐3p expression. (A) Proliferation of human dermal fibroblasts (WS1) after 48 h of heat stress (HS) and subsequent recovery. (B) Migration ability of fibroblasts following HS treatment. (C) mRNA expression levels of fibrosis‐related markers (collagen I, α‐SMA, TGF‐β1) in HS‐treated fibroblasts. (D) Western blot analysis of collagen I, α‐SMA, and TGF‐β1 protein expression in fibroblasts after HS exposure. (E) Quantification of protein expression levels relative to GAPDH. (F) miR‐148a‐3p expression in fibroblasts after HS exposure. (TT: Thermal treatment; ***p < 0.001 relative to CK by one‐way ANOVA.).

Human fibroblasts exhibited activation after recovery from heat stress, which significantly affected cell function. Cells proliferate slowly after 48 h of heat stress, and their proliferative capacity is significantly up‐regulated after 48 h (Figure 2A). In addition, fibroblasts treated with heat stress for 48 h significantly enhanced cell migration capacity (Figure 2B). Consistently, the mRNA expression levels of fibrosis‐related markers, including collagen I, α‐SMA, and TGF‐β1, were significantly upregulated (Figure 2C), which was further confirmed at the protein level by Western blot analysis (Figure 2D,E). The expression level of miR‐148a‐3p in human dermal fibroblasts (WS1) was significantly decreased at 48 h after heat stress (Figure 2F). Based on the above results, we used 48 h after heat stress for subsequent experimental heat treatment.

### 3.4 miR‐148a‐3p Modulates Heat‐Stressed Fibroblast Activity

**FIGURE 3:**
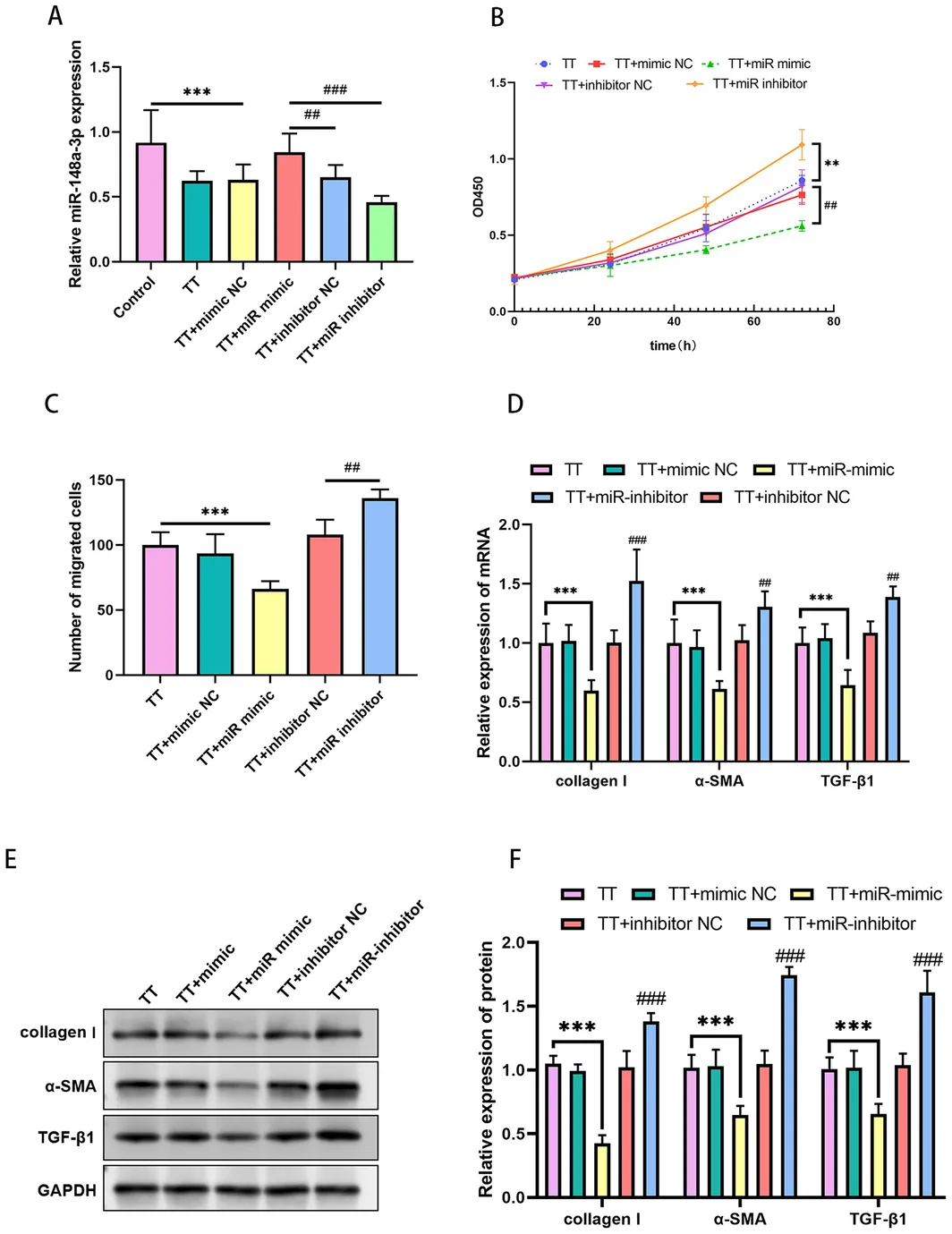
miR‐148a‐3p regulates heat‐stressed fibroblast activity. (A) The decrease in miR‐148a‐3p expression after heat stress can be restored by transfer to its mimic, while the inhibitor further reduces the expression. (B) Cell proliferation was assessed by CCK‐8 assay at 24, 48, and 72 h posttransfection. (C) Quantification of migrated cells from the Transwell assay. (D) mRNA expression levels of fibrotic markers (collagen I, α‐SMA, and TGF‐β1) were determined by qRT‐PCR. (E) Western blot analysis of collagen I, α‐SMA, and TGF‐β1 protein expression. (F) Quantification of protein expression levels relative to GAPDH. (TT: Thermal treatment; ***p < 0.001, **p < 0.01 relative to CK or TT, ^##^ *p* < 0.05, ^###^ *p* < 0.01 relative to TT + mimic NC or TT + inhibitor NC, by one‐way ANOVA).

Transfection with miR‐148a‐3p mimics restored heat stress‐induced downregulation of miR‐148a‐3p to near‐normal levels, while inhibitor treatment further reduced its expression (Figure 3A). Functional assays demonstrated that miR‐148a‐3p overexpression significantly attenuated heat stress‐induced cell proliferation and migration, whereas miR‐148a‐3p inhibition enhanced these effects (Figure 3B,C). Furthermore, miR‐148a‐3p negatively regulated the mRNA expression of fibrotic markers, including collagen I, α‐SMA, and TGF‐β1 (Figure 3D). This negative regulation was further confirmed at the protein level by Western blot analysis (Figure 3E,F).

### 3.5 miR‐148a‐3p Directly Targets MEOX2 and Mediates the Post burn Fibrosis Response

**FIGURE 4:**
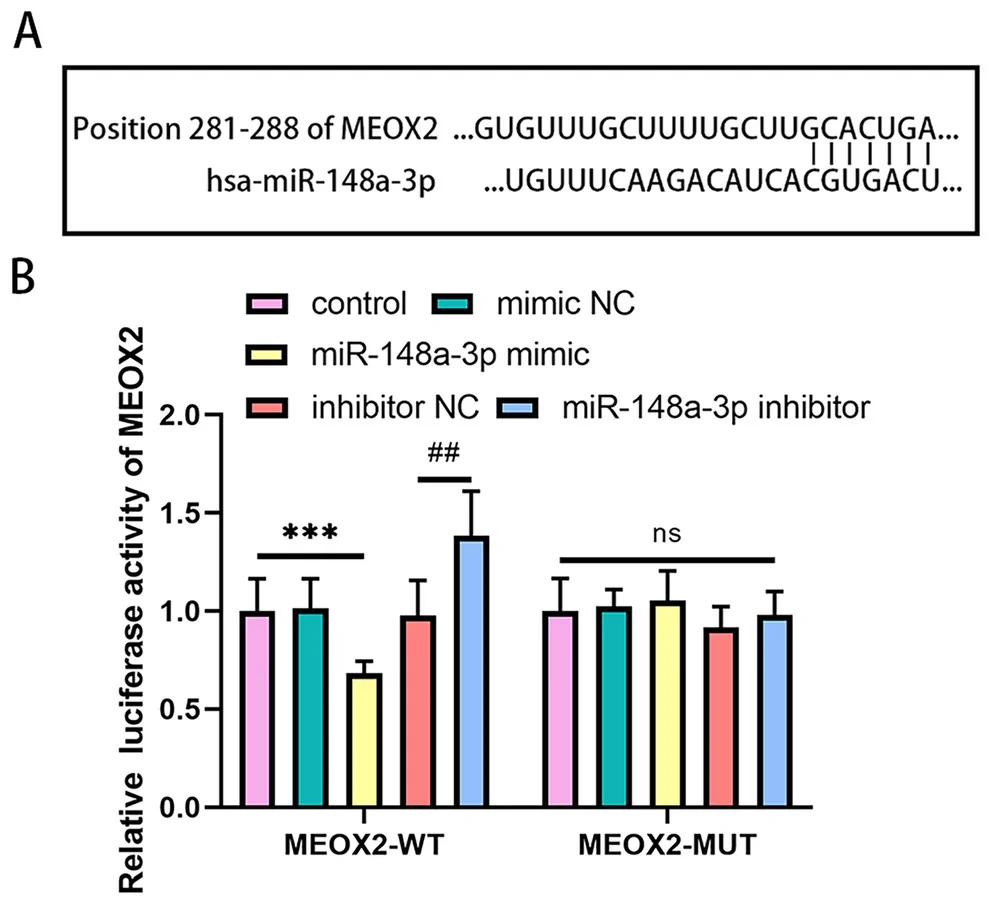
miR‐148a‐3p directly targets MEOX2. (A) Predicted binding site. (B) Luciferase assay validation. (***p < 0.001 relative to CK, ^##^ *p* < 0.05 relative to inhibitor by one‐way ANOVA).

The binding site of miR‐148a‐3p to MEOX2 was predicted by the TargetScanHuman database (Figure 4A). Dual luciferase reporter assays showed that miR‐148a‐3p overexpression significantly inhibited the luciferase activity of MEOX2 wild‐type (WT) vector in human skin fibroblasts under heat stress (Figure 4B).

### 3.6 miR‐148a‐3p Inhibits Skin Fibroblast Proliferation, Migration and Fibrosis by Targeting MEOX2

**FIGURE 5:**
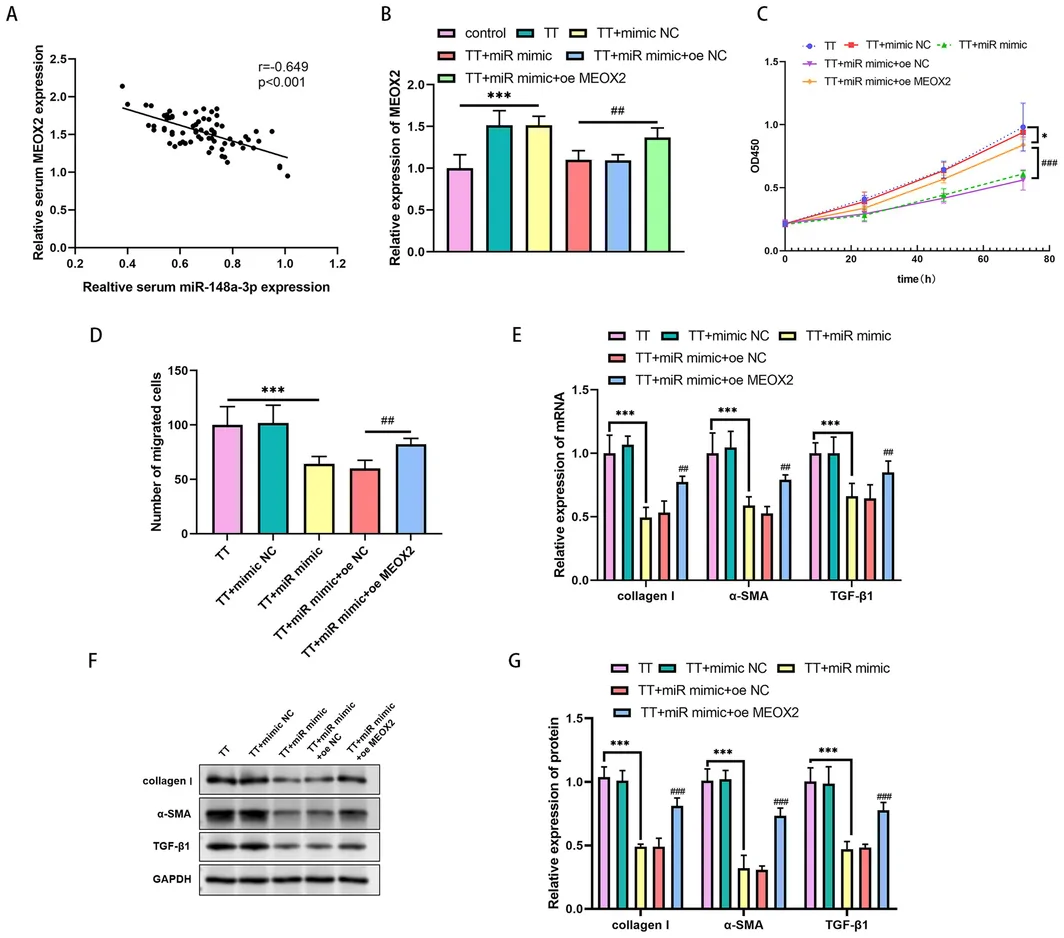
miR‐148a‐3p regulates cellular functions and fibrosis via MEOX2. (A) Negative correlation between miR‐148a‐3p and MEOX2 in patient serum. (B) MEOX2 expression under heat stress (HS) and miR‐148a‐3p mimic transfection. (C, D) Effects of miR‐148a‐3p/MEOX2 on fibroblast proliferation and migration. (E) mRNA expression levels of fibrotic markers (collagen I, α‐SMA, and TGF‐β1) determined by qRT‐PCR. (F) Western blot analysis of collagen I, α‐SMA, and TGF‐β1 protein expression. (G) Quantification of protein expression levels relative to GAPDH (TT: Thermal treatment, oe NC: Overexpression negative control, oe MEOX2: Overexpression MEOX2; ***p < 0.001, *p < 0.05 relative to CK or TT, ^##^ *p* < 0.05 relative to TT + mimic NC + oe NC by one‐way ANOVA).

Correlation analysis of serum samples from clinical patients revealed a significant negative correlation between miR‐148a‐3p and MEOX2 expression levels in burn patients (*r* = −0.649, *p* < 0.001; Figure 5A). The same results were shown at the cellular level, where heat stress significantly upregulated MEOX2 expression, while transfection of miR‐148a‐3p mimics significantly inhibited this effect. Notably, subsequent MEOX2 overexpression restored its expression levels (Figure 5B). Functional analysis showed that miR‐148a‐3p significantly attenuated cell proliferation by targeting MEOX2 overexpression (Figure 5C). Migration assays further confirmed that the miR‐148a‐3p/MEOX2 regulatory axis effectively inhibited cell migration (Figure 5D). Moreover, miR‐148a‐3p significantly downregulated the mRNA expression levels of fibrotic markers (including collagen I, α‐SMA, and TGF‐β1) through MEOX2 regulation (Figure 5E), which was further confirmed at the protein level by Western blot analysis (Figure 5F,G).

## 4 Discussion

Burn scar hyperplasia is a common pathological healing reaction after deep burns, manifested as abnormal thickening, bulging, redness of the skin, accompanied by itching, pain and dysfunction, and its pathological essence is that the overactivation of fibroblasts leads to excessive deposition of collagen fibers and accumulation of myofibroblasts, accompanied by persistent inflammation and abnormal angiogenesis [17, 18]. Currently, no uniformly effective treatment exists for pathological scarring in clinical practice. The mechanism likely involves sustained activation of the TGF‐β1/Smad pathway, promoting fibroblast‐to‐myofibroblast differentiation and excessive collagen I secretion, leading to extracellular matrix deposition and scar contracture. Previous studies indicate that miRNA regulation plays an important role in scar‐related fibrosis, particularly in pathological skin scarring [19]. Specific miRNAs including miR‐29 [20], miR‐1343 [21] and miR‐195 [22] have been demonstrated to be closely associated with fibrous hyperplasia. miR‐148a‐3p, as a miRNA associated with fibrotic diseases, is abnormally expressed in burn tissues and involved in the fibrosis process of the disease [8]. Our study revealed significantly decreased miR‐148a‐3p expression in burn patients, with the lowest levels in scar tissue, suggesting its role in pathological scarring. Further analysis showed that miR‐148a‐3p showed significant predictive value for scar hyperplasia and served as an independent diagnostic marker when combined with burn depth. Postburn scar pathogenesis involves multiple factors including wound depth, inflammation, fibroblast activation, and extracellular matrix deposition [23]. In this cohort, keloid hyperplasia incidence reached 76.4%, confirming its prevalence as a postburn complication. Our findings position miR‐148a‐3p as both a potential diagnostic marker and a promising therapeutic target for early keloid intervention.

To verify the potential cause of miR‐148a‐3p in scarring hyperplasia, we constructed a model of human skin fibroblast burns. The human skin fibroblast line (WS1) is an immortalized fibroblast cell line derived from the normal human fetal dermis, which is widely used in the research of skin fibrosis diseases due to its stable proliferative ability and typical fibroblast characteristics [24]. The key pathological features of scarring can be reproduced by profibrotic factors such as TGF‐β1, including upregulation of α‐smooth muscle actin expression, excessive deposition of Type I collagen, and inhibition of matrix metalloproteinase activity [25, 26]. As an important in vitro research model, WS1 cells have been used to elucidate myofibroblast differentiation mechanisms, evaluate the efficacy of antifibrotic drugs, and study the regulation of inflammatory‐fibrotic networks [27]. In our cell model, a similar situation was observed in burn patients, where the expression of miR‐148a‐3p was observed in human skin fibroblasts after heating, and its expression level gradually decreased with the increase in time after heating. To verify whether burns result in scar hyperplasia by increasing levels of fibrosis, we tested the relevant cytokines. Scar hyperplasia is a pathological repair process primarily mediated by activated fibroblasts and their transformed myofibroblasts [28]. Moderate scar formation aids in wound closure and restoration of tissue integrity, while excessive fibrotic responses can lead to abnormal extracellular matrix deposition and even accelerate the progression of pathological scarring [29]. These activated fibroblasts secrete in large quantities key fibrotic factors such as Collagen I, α‐SMA, and TGF‐β1, which exacerbate scar hyperplasia through multiple pathways: Collagen I is a key indicator of skin fibrosis. Normally, it maintains a 4:1 equilibrium ratio with Type III collagen, but it is over‐deposited in the event of fibrosis, resulting in disproportionality and tissue stiffness [30]. At the same time, collagen fibers thicken and undergo abnormal cross‐linking, further reducing skin elasticity. In keloids, gene and protein expression levels of Collagen I can be as high as 3–5 times that of normal skin, and this significant upregulation is the core molecular basis of the pathological increase in scar hardness [31, 32]. α‐SMA (α‐smooth muscle actin) is a specific marker for myofibroblasts and plays a key role in tissue repair and fibrosis. When tissue is damaged, fibroblasts are transformed into α‐SMA‐positive myofibroblasts stimulated by factors such as TGF‐β, which promotes wound healing. However, sustained activation can lead to excessive contraction and deposition of the extracellular matrix, leading to fibrotic lesions (e.g., keloid contracture and tissue sclerosis). Therefore, α‐SMA levels can directly reflect the degree of fibrosis and are important targets for diagnosis and treatment [33, 34]. TGF‐β1 (transforming growth factor‐β1) is a key profibrotic cytokine that plays a central role in scarring. It leads to excessive deposition of extracellular matrix by activating fibroblasts, promoting collagen synthesis, and inhibiting matrix metalloproteinase activity. At the same time, fibroblasts are induced to differentiate into myofibroblasts, and the tissue contraction ability is enhanced. In pathological scars, the sustained high expression of TGF‐β1 hinders myofibroblast apoptosis and imbalances collagen metabolism, ultimately leading to scar thickening and contracture [35, 36]. The interaction of these factors creates a positive feedback loop that amplifies the fibrotic response, which ultimately leads to scarring. The same results were shown in our burn model, and the expression of effector molecules in these three fibrosis processes was significantly increased by increasing the expression of miR‐148a‐3p and significantly increasing the expression of these three fibrosis processes by increasing the expression of miR‐148a‐3p.

At the same time, the fibrosis process significantly affects cellular function through complex molecular mechanisms. In terms of proliferation, factors such as TGF‐β1 can both stimulate fibroblast proliferation and inhibit epithelial cell growth, resulting in proliferative imbalance [37]. In terms of migration function, although EMT transformation and matrix remodeling promote cell movement, excessive collagen deposition forms a physical barrier [38], this dysfunction can hinder tissue repair and promote fibrosis development. Our results showed that increasing miR‐148a‐3p expression inhibited cell proliferation and migration under the model conditions, while translating to the miR‐148a‐3p inhibitor significantly increased the proliferation and migration function of the model. To investigate the mechanism of miR‐148a‐3p in burn‐induced scar hyperplasia, we conducted a dual‐luciferase assay targeting its potential downstream target, MEOX2. The results demonstrated that miR‐148a‐3p directly binds to MEOX2 and significantly modulates its expression level in the burn model. Furthermore, rescue experiments revealed that MEOX2 overexpression reversed the cellular effects induced by miR‐148a‐3p, including the upregulation of fibrosis‐related factors. These findings indicate that the miR‐148a‐3p/MEOX2 regulatory axis plays a critical role in burn‐related scar hyperplasia.

This study has some limitations despite revealing miR‐148a‐3p's regulatory role in postburn scar formation via MEOX2 targeting. The sample size requires expansion for more reliable conclusions, and the crosstalk between miR‐148a‐3p/MEOX2 and pathways like TGF‐β/Smad needs further exploration. Additionally, the fetal origin of WS1 cells may limit their relevance to adult scarring, warranting future validation using clinical samples. Furthermore, the 6‐month follow‐up period is relatively short, given that hypertrophic scars may continue to evolve both clinically and biologically over a considerably longer timeframe. In the absence of extended longitudinal data, this constraint should be explicitly acknowledged as a limitation of the present study. Moreover, it should be emphasized that the WS1 fibroblast cell line, while useful for preliminary mechanistic investigations, does not fully recapitulate the biological behavior of adult dermal fibroblasts and is even less representative of fibroblasts involved in pathological scar fibrosis. Therefore, it cannot be considered fully representative of the biological processes occurring in burned tissue and the subsequent development of hypertrophic scarring, and future validation using primary adult dermal fibroblasts or scar‐derived fibroblasts from clinical samples is warranted.

## 5 Conclusion

This study reveals the critical regulatory role of miR‐148a‐3p in postburn scar formation, demonstrating its potential as both a diagnostic biomarker and therapeutic target. By targeting MEOX2 to modulate fibroblast activity, our findings provide novel insights into the mechanisms underlying postburn fibrosis. Future research should further explore the clinical applications of miR‐148a‐3p, including the development of molecular‐based early diagnostic approaches and targeted therapeutic strategies, offering new possibilities for improving burn patient outcomes.

## Ethics Statement

The study protocol was approved by The Ethics Committee of The Second Affiliated Hospital of Shandong First Medical University and followed the principles outlined in the Declaration of Helsinki. In addition, informed consent has been obtained from the participants involved.

## Conflicts of Interest

The authors declare no conflicts of interest.

## Data Availability

Data can be shared upon reasonable request by the corresponding author.

## References

[1] C. C. Finnerty , M. G. Jeschke , L. K. Branski , J. P. Barret , P. Dziewulski , and D. N. Herndon , “Hypertrophic Scarring: The Greatest Unmet Challenge After Burn Injury,” Lancet (London, England) 388, no. 10052 (2016): 1427–1436. 27707499 10.1016/S0140-6736(16)31406-4 PMC5380137

[2] L. Jiang , Y. Deng , W. Li , and Y. Lu , “Arctigenin Suppresses Fibroblast Activity and Extracellular Matrix Deposition in Hypertrophic Scarring by Reducing Inflammation and Oxidative Stress,” Molecular Medicine Reports 22, no. 6 (2020): 4783–4791. 33174021 10.3892/mmr.2020.11539 PMC7646887

[3] E. N. Gangemi , D. Gregori , P. Berchialla , et al., “Epidemiology and Risk Factors for Pathologic Scarring After Burn Wounds,” Archives of Facial Plastic Surgery 10, no. 2 (2008): 93–102. 18347236 10.1001/archfaci.10.2.93

[4] D. P. Bartel , “MicroRNAs: Target Recognition and Regulatory Functions,” Cell 136, no. 2 (2009): 215–233. 19167326 10.1016/j.cell.2009.01.002 PMC3794896

[5] G. Liu , A. Friggeri , Y. Yang , et al., “miR‐21 Mediates Fibrogenic Activation of Pulmonary Fibroblasts and Lung Fibrosis,” Journal of Experimental Medicine 207, no. 8 (2010): 1589–1597. 20643828 10.1084/jem.20100035 PMC2916139

[6] M. G. Eissa and C. M. Artlett , “The MicroRNA miR‐155 Is Essential in Fibrosis,” Non‐Coding RNA 5, no. 1 (2019): 23. 30871125 10.3390/ncrna5010023 PMC6468348

[7] L. Chao , Z. Hua‐Yu , B. Wen‐Dong , et al., “miR‐96 Promotes Collagen Deposition in Keloids by Targeting Smad7,” Experimental and Therapeutic Medicine 17, no. 1 (2019): 773–781. 30651862 10.3892/etm.2018.7008 PMC6307430

[8] P. Liang , C. Lv , B. Jiang , et al., “MicroRNA Profiling in Denatured Dermis of Deep Burn Patients,” Burns 38, no. 4 (2012): 534–540. 22360957 10.1016/j.burns.2011.10.014

[9] W. Huang , F. Huang , R. Zhang , and H. Luo , “LncRNA Neat1 Expedites the Progression of Liver Fibrosis in Mice Through Targeting miR‐148a‐3p and miR‐22‐3p to Upregulate Cyth3,” Cell Cycle (Georgetown, Texas) 20, no. 5–6 (2021): 490–507. 10.1080/15384101.2021.1875665 PMC8018424 33550894

[10] Q. Jiang , J. Zhao , Q. Jia , et al., “MiR‐148a‐3p Within HucMSC‐Derived Extracellular Vesicles Suppresses Hsp90b1 to Prevent Fibroblast Collagen Synthesis and Secretion in Silica‐Induced Pulmonary Fibrosis,” International Journal of Molecular Sciences 24, no. 19 (2023): 14477. 37833927 10.3390/ijms241914477 PMC10572270

[11] Q. Jiang , F. Ning , Q. Jia , et al., “MiR‐148a‐3p Loaded Human Umbilical Cord Mesenchymal Stem Cell‐Derived Extracellular Vesicles Alleviates Silica‐Induced Pulmonary Fibrosis by Inhibiting β‐Catenin Signaling,” International Journal of Nanomedicine 20 (2025): 4319–4336. 40230541 10.2147/IJN.S506542 PMC11994480

[12] X. Bai , J. Li , L. Li , et al., “Extracellular Vesicles From Adipose Tissue‐Derived Stem Cells Affect Notch‐miR148a‐3p Axis to Regulate Polarization of Macrophages and Alleviate Sepsis in Mice,” Frontiers in Immunology 11 (2020): 1391. 32719678 10.3389/fimmu.2020.01391 PMC7347748

[13] M. Noizet , E. Lagoutte , M. Gratigny , et al., “Master Regulators in Primary Skin Fibroblast Fate Reprogramming in a Human Ex Vivo Model of Chronic Wounds,” Wound Repair and Regeneration 24, no. 2 (2016): 247–262. 26663515 10.1111/wrr.12392

[14] Q. Wang , T. Li , Y. He , and C. Rao , “MEOX2 Participates in Hepatic Stellate Cells‐Induced Liver Fibrosis by Regulating the PI3K/AKT Signaling Pathway,” Discovery Medicine 36, no. 185 (2024): 1199–1209. 38926106 10.24976/Discov.Med.202436185.110

[15] C. Xie , K. Shi , X. Zhang , J. Zhao , and J. Yu , “MiR‐1908 Promotes Scar Formation Post‐Burn Wound Healing by Suppressing Ski‐Mediated Inflammation and Fibroblast Proliferation,” Cell and Tissue Research 366, no. 2 (2016): 371–380. 27256397 10.1007/s00441-016-2434-6

[16] R. H. Cunnington , J. M. Northcott , S. Ghavami , et al., “The Ski‐Zeb2‐Meox2 Pathway Provides a Novel Mechanism for Regulation of the Cardiac Myofibroblast Phenotype,” Journal of Cell Science 127, no. Pt 1 (2014): 40–49. 24155330 10.1242/jcs.126722

[17] Y. Wang , J. Beekman , J. Hew , et al., “Burn Injury: Challenges and Advances in Burn Wound Healing, Infection, Pain and Scarring,” Advanced Drug Delivery Reviews 123 (2018): 3–17. 28941987 10.1016/j.addr.2017.09.018

[18] P. D'Arpa and K. P. Leung , “Pharmaceutical Prophylaxis of Scarring With Emphasis on Burns: A Review of Preclinical and Clinical Studies,” Adv Wound Care (New Rochelle) 11, no. 8 (2022): 428–442. 33625898 10.1089/wound.2020.1236 PMC9142134

[19] M. Li , D. W. Liu , and W. Lei , “Advances in the Research of Effects of Competing Endogenous RNAs and Their Regulatory Networks in Pathological Scars of Skin,” Zhonghua Shao Shang Za Zhi = Zhonghua Shaoshang Zazhi = Chinese Journal of Burns 35, no. 9 (2019): 701–704. 31594191 10.3760/cma.j.issn.1009-2587.2019.09.011

[20] M. Wang , Z. Huo , X. He , et al., “The Role of MiR‐29 in the Mechanism of Fibrosis,” Mini Reviews in Medicinal Chemistry 23, no. 19 (2023): 1846–1858. 37018517 10.2174/1389557523666230328125031

[21] L. R. Stolzenburg and A. Harris , “Microvesicle‐Mediated Delivery of miR‐1343: Impact on Markers of Fibrosis,” Cell and Tissue Research 371, no. 2 (2018): 325–338. 29022142 10.1007/s00441-017-2697-6 PMC5809304

[22] D. M. Wang , J. J. Jin , L. M. Tian , and Z. Zhang , “MiR‐195 Promotes Myocardial Fibrosis in MI Rats via Targeting TGF‐β1/Smad,” Journal of Biological Regulators and Homeostatic Agents 34, no. 4 (2020): 1325–1332. 32914608 10.23812/20-201-A

[23] G. G. Gauglitz , H. C. Korting , T. Pavicic , T. Ruzicka , and M. G. Jeschke , “Hypertrophic Scarring and Keloids: Pathomechanisms and Current and Emerging Treatment Strategies,” Molecular Medicine (Cambridge, Mass) 17, no. 1–2 (2011): 113–125. 20927486 10.2119/molmed.2009.00153 PMC3022978

[24] X. Li , N. Li , Y. Wang , Q. Han , and B. Sun , “Research Progress of Fibroblasts in Human Diseases,” Biomolecules 14, no. 11 (2024): 1478. 39595654 10.3390/biom14111478 PMC11591654

[25] L. Moretti , J. Stalfort , T. H. Barker , and D. Abebayehu , “The Interplay of Fibroblasts, the Extracellular Matrix, and Inflammation in Scar Formation,” Journal of Biological Chemistry 298, no. 2 (2022): 101530. 34953859 10.1016/j.jbc.2021.101530 PMC8784641

[26] R. Ţuţuianu , A. M. Roşca , G. Florea , et al., “Heterogeneity of Human Fibroblasts Isolated From Hypertrophic Scar,” Romanian Journal of Morphology and Embryology 60, no. 3 (2019): 793–802. 31912089

[27] M. H. Liao , S. S. Liu , I. C. Peng , F. J. Tsai , and H. H. Huang , “The Stimulatory Effects of alpha1‐Adrenergic Receptors on TGF‐beta1, IGF‐1 and Hyaluronan Production in Human Skin Fibroblasts,” Cell and Tissue Research 357, no. 3 (2014): 681–693. 24844469 10.1007/s00441-014-1893-x

[28] M. B. van der Wal , J. F. Vloemans , W. E. Tuinebreijer , et al., “Outcome After Burns: An Observational Study on Burn Scar Maturation and Predictors for Severe Scarring,” Wound Repair and Regeneration 20, no. 5 (2012): 676–687. 22985039 10.1111/j.1524-475X.2012.00820.x

[29] J. Q. Coentro , E. Pugliese , G. Hanley , M. Raghunath , and D. I. Zeugolis , “Current and Upcoming Therapies to Modulate Skin Scarring and Fibrosis,” Advanced Drug Delivery Reviews 146 (2019): 37–59. 30172924 10.1016/j.addr.2018.08.009

[30] L. Lin‐Hui , Z. Yuan‐Yuan , L. Ming‐Yu , et al., “Recombinant Human Collagen Type III Improves Hypertrophic Scarring by Regulating the Ratio of Type I/III Collagen,” Journal of Burn Care & Research: Official Publication of the American Burn Association 45, no. 5 (2024): 1269–1273. 38477626 10.1093/jbcr/irae040

[31] F. Syed , E. Ahmadi , S. A. Iqbal , S. Singh , D. A. McGrouther , and A. Bayat , “Fibroblasts From the Growing Margin of Keloid Scars Produce Higher Levels of Collagen I and III Compared With Intralesional and Extralesional Sites: Clinical Implications for Lesional Site‐Directed Therapy,” British Journal of Dermatology 164, no. 1 (2011): 83–96. 20849516 10.1111/j.1365-2133.2010.10048.x

[32] R. M. Biggs and A. D. Bradshaw , “Organ Fibrosis: Beyond Collagen I Expression. Fibroblast Phenotype and Basement Membrane Proteins,” American Journal of Physiology. Cell Physiology 328, no. 6 (2025): C2023–c2031. 40353336 10.1152/ajpcell.00077.2025 PMC12168137

[33] I. Cardoso‐Lezama , E. Ramos‐Tovar , J. Arellanes‐Robledo , et al., “Serum α‐SMA Is a Potential Noninvasive Biomarker of Liver Fibrosis,” Toxicology Mechanisms and Methods 34, no. 1 (2024): 13–19. 37528633 10.1080/15376516.2023.2244061

[34] H. Ding , J. Chen , J. Qin , R. Chen , and Z. Yi , “TGF‐β‐Induced α‐SMA Expression Is Mediated by C/EBPβ Acetylation in Human Alveolar Epithelial Cells,” Molecular Medicine (Cambridge, Mass) 27, no. 1 (2021): 22. 33663392 10.1186/s10020-021-00283-6 PMC7934236

[35] K. K. Kim , D. Sheppard , and H. A. Chapman , “TGF‐β1 Signaling and Tissue Fibrosis,” Cold Spring Harbor Perspectives in Biology 10, no. 4 (2018): a022293. 28432134 10.1101/cshperspect.a022293 PMC5880172

[36] S. Hata , K. Okamura , M. Hatta , H. Ishikawa , and J. Yamazaki , “Proteolytic and Non‐Proteolytic Activation of Keratinocyte‐Derived Latent TGF‐β1 Induces Fibroblast Differentiation in a Wound‐Healing Model Using Rat Skin,” Journal of Pharmacological Sciences 124, no. 2 (2014): 230–243. 24492413 10.1254/jphs.13209fp

[37] Y. Zhang , P. B. Alexander , and X. F. Wang , “TGF‐β Family Signaling in the Control of Cell Proliferation and Survival,” Cold Spring Harbor Perspectives in Biology 9, no. 4 (2017): a022145. 27920038 10.1101/cshperspect.a022145 PMC5378054

[38] S. Rhee , “Fibroblasts in Three Dimensional Matrices: Cell Migration and Matrix Remodeling,” Experimental & Molecular Medicine 41, no. 12 (2009): 858–865. 19745603 10.3858/emm.2009.41.12.096 PMC2802681

